# Is %**Δ**SUVmax a Useful Indicator of Survival in Patients with Advanced Nonsmall-Cell Lung Cancer?

**DOI:** 10.1155/2013/910957

**Published:** 2013-10-09

**Authors:** Angelina Cistaro, Natale Quartuccio, Alireza Mojtahedi, Piercarlo Fania, Pier Luigi Filosso, Mariapaola Cucinotta, Alfredo Campennì, Umberto Ficola, Sergio Baldari

**Affiliations:** ^1^Positron Emission Tomography Centre, IRMET S.p.A., Euromedic, V.O. Vigliani 89, 10136 Turin, Italy; ^2^PET Pediatric AIMN InterGroup, 10136 Turin, Italy; ^3^Institute of Cognitive Sciences and Technologies, National Research Council, 00185 Rome, Italy; ^4^Department of Biomedical Sciences and of Morphological and Functional Images, Nuclear Medicine Unit, University of Messina, 98125 Messina, Italy; ^5^Nuclear Medicine Service, Memorial Sloan-Kettering Cancer Center, New York, NY 10021, USA; ^6^Department of Thoracic Surgery, S. Giovanni Battista Hospital, 10126 Turin, Italy; ^7^Department of Nuclear Medicine, La Maddalena Hospital, 90146 Palermo, Italy

## Abstract

*Purpose*. To investigate the impact of the maximum standardized uptake value (SUVmax), size of primary lung lesion, and %ΔSUVmax on outcome (overall survival (OS) and 2-year disease-free survival (2-year DFS)) of patients with advanced nonsmall-cell lung cancer (NSCLC). *Materials and Methods*. 86 stage III-IV NSCLC patients underwent 18 F-FDGPET/CT, before and after chemotherapy, and were classified into subgroups according to the response criteria of the European Organization for Research and Treatment of Cancer. SUVmax values and tumor size with the best prognostic significance were searched. Correlation between the SUVmax value and the initial response to therapy (best response) and the relationship between %ΔSUVmax and OS were assessed. *Results*. In patients in PD (20/86), the average pretreatment SUVmax was 11.8 ± 5.23, and the mean size of the primary lesion was 43.35 mm ± 16.63. In SD, PR, and CR patients (66/86), the average pretreatment SUVmax was 12.7 ± 8.05, and the mean size of the primary lesion was 41.6 mm ± 21.15. Correlation was identified only for %ΔSUVmax; patients with PD (ΔSUVmax > +25%) showed a worse OS than patients with ΔSUVmax < +25% (CR, PR, and SD) (*P* = 0.0235). *Conclusions*. In stage III-IV NSCLC, among the assessed factors, only %ΔSUVmax may be considered as a useful prognostic factor.

## 1. Introduction

Among worldwide oncologic causes of death, lung cancer is the leading one [1]. Cross-sectional imaging using computed tomography (CT) combined with positron-emission tomography (PET) provides excellent information about anatomic and metabolic evaluation of malignancies. In order to stage, restage, and evaluate the treatment response in lung cancer, 2-deoxy-2-[18F]fluoro-D-glucose positron-emission tomography (^18^F-FDG PET/CT) is widely used [2, 3]. PET/CT has been utilized in the evaluation of response to treatment, and several studies demonstrated the correlation between decreased metabolic activity of the tumor following treatment and survival. Cisplatin-based chemotherapy is currently a key strand for treatment of advanced (stages III-IV) nonsmall-cell lung cancer (NSCLC). Despite the introduction of modern treatment protocols, prognosis of advanced NSCLC still remains poor, especially in comparison with disease in early stage [1]. While FDG-PET/CT has been demonstrated to provide additional prognostic information for patients with stages I-II NSCLC before treatment [2, 4], it has a limited prognostic role in stages III-IV [3]. However, previous study has demonstrated that FDG-PET/CT can be considered as a helpful tool in assessing therapeutic efficacy of chemotherapy in NSCLC [5]. According to the response criteria recommended by the European Organization for Research and Treatment of Cancer (EORTC), a metabolic response correlates with survival [6]. The early metabolic response, with significant reduction of tracer uptake compared with the baseline study before therapy, has been found predictive of favorable prognosis. Instead a slight reduction or an increased uptake is predictive of treatment failure or disease progression [5–7]. The aim of this study was to identify the possible cutoff values for SUVmax and size of primary lung lesion that have correlation with clinical outcome (OS and DFS) in patients with advanced (stages III-IV) NSCLC. We also evaluated the correlation between the pretreatment SUVmax and the initial response to therapy (best response) and between %ΔSUVmax (the change between SUVmax before and after chemotherapy) and outcome.

## 2. Materials and Methods

Eighty-six patients (67 M and 19 F; M/F ratio = 3.5 : 1; mean age = 63.5 years; range = 37–80) were referred to IRMET Positron Emission Center S.p.A., Euromedic, in Turin, Italy, and Unit of Nuclear Medicine of La Maddalena Hospital in Palermo, Italy, for evaluation of their advanced non-small cell lung cancer. The histotypes were 41 (48%) adenocarcinomas, 16 (19%) squamous cell carcinomas, 9 (10%) large cell carcinomas, and 20 (23%) uncommon histotypes (others) (Table 1).

Patients were staged according to the TNM 7th edition [8] in stages IIIA, IIIB, and IV via information gathered through patient's chart including physical examination, routine blood test, bronchoscopy, contrast-enhanced computed tomography (CT) of the chest and upper abdomen, and brain CT. Survival and death information were retrieved by means of the hospitals databases. The research proposal was approved by Institutional Review Board and Ethics Committee. All patients underwent chemotherapeutic treatment based on cisplatin and taxanes drugs (135 mg of paclitaxel per square meter of body-surface area, administered over a 24-hour period on day 1, followed by 75 mg of cisplatin per square meter on day 2, every three weeks) and two FDG PET/CT whole body scan, one before (T0) and one after treatment (T1: one month). IIIA patients undergoing neoadjuvant chemotherapy and subsequent surgery were excluded from the study. Patients were excluded from the study also in case of (a) poor performance status; (b) Charlson Combined Age-Comorbidity Index ≥ 6; (c) histological diagnosis of “bronchioloalveolar cell carcinoma” (BAC) subtype. PET/CT images were acquired at both centres (Turin and Palermo) on an integrated PET/CT scanner (Discovery ST, General Electric Medical System), 60 minutes after the injection of the 260–420 MBq of ^18^F-FDG, following 6 hours of fasting from the cranial base to the pelvis (total body) in 3D modality. The PET images were reconstructed iteratively on a 128 × 128 matrix. The SUVmax, within a spherical region of interest encompassing the entire volume, was calculated using a GE Xeleris workstation (General Electric Medical System, Milwaukee, WI, USA). The studies were interpreted by experienced nuclear medicine physician at both centers. The anatomical size of lung lesion was measured considering the maximum diameter of the lesion in the three planes [9]. Patients were followed up for 24 months with a frequency of clinical examination of every 3 months during the first year and every 6 months in the second year. A contrast-enhanced CT scan of the thorax was performed in all cases at 6 and 12 months, while the additional PET/CT scans were used in case of suspected morphological findings. At the end of the first-line chemotherapy treatment, the patients were further divided into the following 4 subgroups based on the %ΔSUVmax of the tumor, according to the EORTC response criteria [6]: stable disease (SD) (Δ < −25% to Δ < +25%), partial response (PR) (Δ > −25%), complete response (CR) (complete disappearance), and progressive disease (PD) (Δ > +25%). Overall survival was calculated separately for PET responder patients (SD, PR, and CR) and patients in disease progression (PD). Statistical correlation was searched for the following pairs of parameters: pretreatment SUVmax and outcome (OS and DFS), size and outcome (OS and DFS), pretreatment SUV max and best response, and size and best response using Student's *t*-test (the hypothesis was considered significant if *P* ≤ 0.05). Furthermore the correlation between %ΔSUVmax and outcome (OS and DFS) was assessed using the chi-square test (*χ* ^2^-test).

## 3. Results

**Figure 1:**
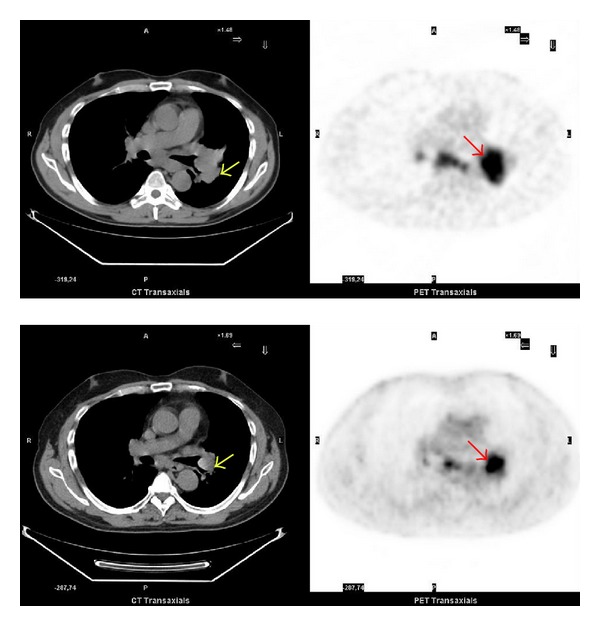
Pre- and posttreatment ^18^F-FDG PET/CT scan. Top: pretreatment ^18^F-FDG PET/CT axial image: staging on a patient with a left hilar adenocarcinoma (yellow arrow) with a SUVmax = 11 (red arrow) and diameter of 40 mm. Abnormal uptake present also in a subcarinal lymph node. Bottom: posttreatment ^18^F-FDG PET/CT axial image: the study was performed one month later, at the end of the first-line chemotherapy. Note the decrease of the ^18^F-FDG uptake (red arrow) in the primary lung lesion (SUVmax = 8) and also an important reduction of the lesion size (yellow arrow).

Thirteen patients out of 86 patients (15%) were at stage IIIA, 18 (21%) at stage IIIB, and 55 (64%) at stage IV. At the staging, SUVmax and diameter of the primitive neoplastic lung lesion were evaluated in each patient. Patients showed an average baseline SUVmax of 12.5 ± 7.43 and a mean diameter of the primary lesion of 42 ± 20 mm. At the end of the first-line chemotherapy, according to EORTC criteria, 20/86 patients were in PD, 24/86 were SD, 35/86 had a PR, and 7/86 showed a CR (Figure 1).

Thirty-seven out of 86 patients were deceased at the follow-up time of 24 months (OS = 57%). In the 20 (23%) patients in progressive disease (PD), the recorded average pretreatment SUVmax was 11.8 ± 5.23, and the mean size of the primary lesion was 43.35 mm ± 16.63. Thirteen/20 patients had already died at the end of the followup (OS = 35%). In the 66 (77%) patients in SD, PR, or CR, the average value of pretreatment SUVmax was 12.7 ± 8.05, and the mean size of the primary lesion was 41.6 mm ± 21.15 (Table 2).

24/66 patients passed away at the end of the followup (OS = 64%). Using Student's *t*-test, no significant correlation was discovered between the following pairs of parameters: pretreatment SUVmax and outcome, and size and outcome, SUV max and best response, size and best response (*P* = n.s.). Using the *χ* ^2^-test, no SUVmax cutoff with prognostic significance was found (*P* = n.s.). However, analyzing the relationship between ΔSUVmax and overall survival, we found that patients with Δ < +25% (CR, PR, and SD) showed a better overall survival than patients in PD (Δ > +25) (*P* = 0.0235, *χ* ^2^-test).

## 4. Discussion

The quantification of FDG uptake can be assessed by several parameters including standardized uptake value (SUV) or local metabolic rate of glucose. SUVmax still remains the most frequently used in clinical routine. In NSCLC, SUV quantification has been proved to have a role in the prediction of outcome [10–15]. For our group of 86 patients in stages III-IV undergoing chemotherapy, no correlation neither between the pretreatment SUVmax in the primary tumor and survival (OS and DFS) nor between pretreatment SUVmax and best response has been found (*P* = n.s.). Even the attempt to apply the *χ* ^2^-test to identify a SUVmax cut-off value with a powerful impact on the prognosis was unsuccessful. There are very few studies that investigated the SUVmax cut-off value and the prognosis of advanced nonsmall-cell lung cancer, and our data was very similar to those few reported in the literature [3, 16]. Hoang and colleagues divided patients into two subgroups using a median SUV of 11.1 but did not find significant correlation between ^18^F-FDG uptake and median survival (16 months versus 12 months) [3]. The authors explained that the absence of correlation between survival and SUV of the primary tumor for patients in stages III-IV may be a consequence of the metastatic situation in these patients being more important for disease-free and overall survival. Other important variables are the clinical complexity of comorbidity of these patients, the prognostic score, and the side effects of the treatments [17, 18]. In our study, the only parameter that showed a correlation with outcome (OS) was %ΔSUVmax but was only marginally associated. Patients with Δ < +25% (CR, PR, and SD) showed a better overall survival than patients in PD (Δ > +25) (64% versus 35%) (*P* = 0.0235). So far, in a single well-designed study on response assessment in advanced stages (stage IIIB or IV), Weber et al. [5] showed that a reduction of metabolic activity already after 1 cycle of chemotherapy is closely correlated with final outcome of therapy. Fifty-seven patients scheduled to receive platinum-based chemotherapy underwent PET scan before and after the first cycle of therapy. A decrease in SUV of 20% or more after 1 cycle of chemotherapy was associated with a longer time to progression (163 versus 54 d) and longer median OS time (252 versus 151 d). The authors showed also a significantly higher 1-year survival rate in metabolic responders compared with nonresponders (44% versus 10%) [5]. Similarly, Kim and colleagues described the prognostic usefulness of ^18^F-FDG PET after chemotherapy in patients with advanced stages III and IV NSCLC and demonstrated that when the tumor reveals more than 17.85% reduction of %ΔSUVmax the survival could be predicted, but their study was limited at only 19 patients [19]. Further confirmatory data come from the study performed by Mac Manus et al. [20] showing a significantly longer median survival for patients with complete metabolic response than for patients with incomplete metabolic response (31 versus 11 months).

Our study had several limitations including lack of SUV assessment in each single histotype and other more recent metabolic indices, such the Metabolic Tumor Volume.

## 5. Conclusions

Our data suggested that SUVmax has not prognostic predictive value in the advanced NSCLC (III-IV). No useful correlation between SUVmax, tumor size, and clinical outcome of NSCLC patients in stages III-IV was found. In patients with advanced disease, only %ΔSUVmax may be considered as a useful prognostic factor. Additional studies are required to further accurately assess the role of SUVmax in advanced nonsmall-cell lung cancer.

## Figures and Tables

**Table 1:** Characteristics of population.

Number of patients	86
Sex	67 M 19 F
Mean age	63.5 y (range: 37–80)
Histotypes	
Adenocarcinoma	41
Squamous	16
Large cell	9
Others	20

M: male; F: female; y: years.

**Table 2:** Classes of metabolic response to therapy and average values of SUVmax and primitive lung lesion diameter.

Classes of response to therapy	Number	Average SUVmax value	Average primitive lung lesion diameter
CR			
PR	66	12.7 ± 8.05	41.6 mm ± 21.15
SD			
PD	20	11.8 ± 5.23	43.35 mm ± 16.63
